# Editorial: The challenge towards more sustainable lithium ion batteries: from their recycling, recovery and reuse to the opportunities offered by novel materials and cell design

**DOI:** 10.3389/fchem.2024.1421434

**Published:** 2024-05-14

**Authors:** Mirko Magni, Marcello Colledani, Gavin Harper

**Affiliations:** ^1^ Department of Environmental Science and Policy, University of Milan, Milan, Lombardy, Italy; ^2^ Department of Mechanical Engineering, Polytechnic of Milan, Milan, Lombardy, Italy; ^3^ School of Metallurgy and Materials, University of Birmingham, Birmingham, United Kingdom

**Keywords:** lithium-ion batteries, recycling, second-life batteries, electrode materials, sustainability

One of most successful outcomes of the modern applied scientific research in the domain of our energy system transformation are lithium ion batteries (LIBs), efficient everyday components of goods designed to store energy and make it available on request. These electrochemical devices offer high modularity and scalability in terms of power and energy capacity and represent today a fundamental technology to support the energy transition required to reduce the emissions from fossil fuels. The present Research Topic has aimed at collecting some of the more recent efforts facing some thorny issues LIBs still suffer from.

One of the more critical point concerns the management and treatment of spent batteries. The first step for the creation of a circular battery value chain should be the development of standard decisional processes able to efficiently discriminate between two main end-of-life scenarios:

- reusing/repurposing, aimed at extending the life-time of batteries by employing them in, usually, less-demanding applications;
- recycling, by dismantling, sorting, and pre-treating the batteries to refine and recover their valuable materials.

As reviewed by Patel et al., the issue is complex as it spreads over distinct but interrelated levels. From a technical point of view, a codification of the State of Health (SoH) of a cell is fundamental to choose the proper pathway it should follow. The second need regards the regulation of the LIBs economy. Thus, a first urgency is the rationalization and standardization of production (*i.e*., variety of cell types), installation (*i.e.*, variety of battery packs) and performance monitoring (*i.e.*, SoH or other criteria) of these electrochemical devices. It is also necessary to create policies, regulations and standards aimed at supporting the implementation of any future process and infrastructure that should manage, at a global level, the end-of-life of LIBs in an environmentally and economically sustainable way.

When second life approach is not possible (*i.e.*, unsatisfactory SoH), the technological challenge is to develop industrially relevant recycling processes able to recover as many materials as possible from cells to make them feed for the production of new batteries. In this scenario, two requirements strictly coexist: the satisfaction of the stringent recovery threshold levels defined in regulatory frameworks and the economic sustainability (besides the environmental one) of the designed processes. The contribution of Fink et al. focuses on the direct recycling. This approach aims at maintaining the integrity and functionality of the target material (*i.e.*, its added-value) so that it could enter the same production chain at a downstream phase, saving energy (*i.e*., for its production by recycled precursor(s)). As industry transitions to lower value cathode materials, direct recycling is likely to increase in importance. In the article, Authors aims at solving the contamination of black mass (BM) by copper and aluminium residues that prevents any direct reuse. Starting from a synthetic cathode BM, artificially spiked with Al and Cu small sheets, Authors showed how a KOH-based aqueous solution is able to quantitatively dissolve metals without damaging the structural and electrochemical features of the active material. Nonetheless, further improvements are still required as residual surface impurities on the recovered metal oxide negatively affect the performance in full-cell testing.

A second relevant task for the research concerns boosting LIBs performance (*i.e.*, higher-power and longer-lasting) to meet the increasingly stringent demands of the market. The Research Topic collected four articles facing the issue of improving the capacity of lithium-ion cells by substituting graphite with novel anode materials exhibiting better theoretical energy density. Invariably, the challenge is to find candidates that, besides offering low-cost, abundance and environmental friendliness, preserve the good mechanical stability offered by the carbon-based benchmark during the lithiation/delithiation cycles, which ultimately affects the life span of the battery itself.

Liu et al. have developed a tin-based composite that synergistically exploits the features of SnO_2_ (high energy density) with those of carbon-based materials (low volume change upon ingress/egress of Li^+^, high conductivity, high surface area). Authors prepared tin oxide carbon spheres that were then further modified, through a microwave-assisted synthesis, with an outermost layer of graphene (SnO_2_@C/graphene). In half-cell configuration, SnO_2_@C/graphene outperformed commercial graphite, reaching a reversible specific capacity of 820 mAh g^−1^ after 100 cycles at 1 A g^-1^.

Zhao et.al. have focused on silicon aiming at the production of 1D nano-architecture to solve the intrinsic drawbacks of this material. Interestingly, Authors demonstrated that Si nanofibers can be produced through a low-temperature aluminothermic reduction process starting from the clay mineral sepiolite heated at 260°C for 10 h. The presence of layers of Mg-O octahedron in sepiolite structure is crucial to preserve the inherent fibrous structure of the mineral that in turns allows the final material to outperform performance of Si anodes already reported.

Manganese is another valuable candidate. In this Research Topic, manganese is declined into two chemical forms: phosphoselenide and oxide. Shen et al. prepared exfoliated few-layered MnPSe_3_ nanoflakes by ultrasonicating the bulk ternary compound. The resulting 2D thin layer architecture results fundamental to overcome the intrinsic limitations of the bulk material, reaching a specific capacity of 578 mAh g^−1^ after 350 cycles at 0.2 A g^-1^. Nanostructuring is the solution proposed also by Wang et al. that chose Mn_3_O_4_ as the target material. Nanopowder oxide was synthetized starting from a solution of manganese salt, sucrose and H_2_O_2_ through a one-step flame spray pyrolysis method that significantly reduces costs and environmental impact with respect to conventional wet phase-based approaches.

In conclusion, the present Research Topic shines light on some of the efforts done to make LIBs able to satisfy the near future needs along the entire life-cycle, from performance requirements to end-of-life and material recovery and re-use. As a guideline to move towards a greener and more sustainable LIB value-chain, it is desirable that academic research tries to fill two gaps still affecting the present related literature:

i) performing recycling studies on more realistic matrices that, by resembling the feeds of future plants, can provide reliable outcomes for industry, for present and future battery chemistries and structures;
ii) including eco-design approach, in both material selection and cell design/assembly, for the development of the next-generation batteries to reduce the exploitation of critical raw materials (contributing in causing environmental, socio, political and economic issues) and to simplify the recycling processes (first of all, the initial mechanical disassembly stage).

Valorisation of spent LIBs is a challenge that academia can work on, however, to ensure real world impact, there is need for further dialogue between all stakeholders to create an environment that supports the translation of these innovations into practice.

